# Applying GC-MS based serum metabolomic profiling to characterize two traditional Chinese medicine subtypes of diabetic foot gangrene

**DOI:** 10.3389/fmolb.2024.1384307

**Published:** 2024-04-25

**Authors:** Jiawei Feng, Yuqing Wang, Shengmin Xiang, Yun Luo, Yongcheng Xu, Yuzhen Wang, Yemin Cao, Mingmei Zhou, Cheng Zhao

**Affiliations:** ^1^ Shanghai Traditional Chinese Medicine Integrated Hospital, Shanghai University of Traditional Chinese Medicine, Shanghai, China; ^2^ Institute of Interdisciplinary Integrative Medicine Research, Shanghai University of Traditional Chinese Medicine, Shanghai, China

**Keywords:** diabetic foot gangrene, traditional Chinese medicine, subtype characterization, metabolomics, GC-MS

## Abstract

Traditional Chinese medicine (TCM) has a long history and particular advantages in the diagnosis and treatment of diabetic foot gangrene (DFG). Patients with DFG are mainly divided into two subtypes, tendon lesion with edema (GT) and ischemic lesion without edema (GI), which are suitable for different medical strategies. Metabolomics has special significance in unravelling the complexities of multifactorial and multisystemic disorders. This study acquired the serum metabolomic profiles of two traditional Chinese medicine subtypes of DFG to explore potential molecular evidence for subtype characterization, which may contribute to the personalized treatment of DFG. A total of 70 participants were recruited, including 20 with DM and 50 with DFG (20 with GI and 30 with GT). Conventional gas chromatography-mass spectrometry (GC-MS) followed by orthogonal partial least-squares discriminant analysis (OPLS-DA) were used as untargeted metabolomics approaches to explore the serum metabolomic profiles. Kyoto encyclopedia of genes and genomes (KEGG) and MetaboAnalyst were used to identify the related metabolic pathways. Compared with DM patients, the levels of 14 metabolites were altered in the DFG group, which were also belonged to the differential metabolites of GI (13) and GT (7) subtypes, respectively. Among these, urea, α-D-mannose, cadaverine, glutamine, L-asparagine, D-gluconic acid, and indole could be regarded as specific potential metabolic markers for GI, as well as L-leucine for GT. In the GI subtype, D-gluconic acid and L-asparagine are positively correlated with activated partial thromboplastin time (APTT) and fibrinogen (FIB). In the GT subtype, L-leucine is positively correlated with the inflammatory marker C-reactive protein (CRP). Arginine and proline metabolism, glycine, serine and threonine metabolism, phenylalanine, tyrosine and tryptophan biosynthesis are the most important metabolic pathways associated with GI. The main metabolic pathways related to GT include pyrimidine metabolism, glutathione metabolism, biosynthesis of valine, leucine, and isoleucine, as well as valine, serine, and isoleucine with metabolites. The results of this study indicate that patients with different DFG subtypes have distinct metabolic profiles, which reflect the pathological characteristics of each subtype respectively. These findings will help us explore therapeutic targets for DFG and develop precise treatment strategies.

## 1 Introduction

Diabetic foot gangrene (DFG) refers to the most serious lesion in the feet or lower extremities of diabetic patients. Improper treatment of DFG can lead to longitudinal development, or even disability and amputation (Lin et al., 2020). Its pathological manifest is mainly focus on peripheral vascular infections and neuropathy caused by hyperglycemia. At present, the annual incidence of diabetic foot in diabetic patients over 50 years old in China is 8.1%, and the annual recurrence rate and annual fatality rate are as high as 31.6% and 14.4%, respectively. The current methods of treating DFG mainly include glycemic control, antibiotic therapy of the infected wounds, enhancing vascularization, debridement, application of wound dressings, negative pressure wound therapy, maggot therapy, use of growth factors, and skin substitutes (Lim et al., 2017). Although these treatments are effective, some are expensive and have negative side effects.

Traditional Chinese medicine (TCM) is a complementary and alternative medicine with individualized diagnosis and treatment system (Rioux, 2019). Holistic concept and dialectical treatment are two basic characteristics of TCM, in which syndrome differentiation (Zheng in Chinese) profiles an overall body condition of patients (symptoms, feelings, tongue appearance, pulse waves, etc.) and plays an important role in TCM diagnosis and treatment (Bao et al., 2010; Shan et al., 2014). TCM syndromes provide different standard of classification, which may contribute to the early and precise diagnosis of diseases (Jiang et al., 2012). Different from several classification systems that assess the severity of diabetic foot lesion by encompass different characteristics of ulcer, such as ulcer size, depth, local ischemia, infection and neuropathy, the clinical classification of DGF in TCM is mainly based on “ZHENG” (syndrome) differentiation. For the treatment of diseases with external traumatic wound, such as DFG, syndrome must be a whole combination of holistic and local syndrome differentiation, with the latter accounting for a greater weight. Most DFG patients have the dampness-heat syndrome (DHS), the trauma of which is characterized by edema, degeneration, and decay of tendon tissue, as well as the formation of penetrating ulcers along the tendon accompanied by extensive exudation. This subtype mainly characterized by tendon lesion with edema is called “JIN JU” in Chinese (GT). The clinical manifestations were mainly local infection, often accompanied by peripheral neuropathy. DHS is mostly reflected by the changes in abnormal immune function and inflammatory factors, such as C-reactive protein (CRP). Another major subtype of DFG, known as “TUO JU” in Chinese (GI), is characterized by blood stasis and presents as ischemic lesions without edema. The characteristics of necrosis are also significantly different from those of severe infections caused by tendon degeneration and necrosis in GT patients, mainly characterized by dry gangrene with localized blackening of the epidermis. Due to insufficient blood supply, systemic immunologic cells and factors are difficult to reach the local traumatic area, causing corresponding inflammatory reactions, thus the infection in local traumatic are is relatively mild in GI patients. Most patients with blood stasis had changes related to coagulation and plasma viscosity. As a crucial indictor for both whole blood and plasma viscosities, fibrinogen (FIB) is the core indicator to diagnose blood stasis (Rasyid et al., 2019; Sun et al., 2022; Valeanu et al., 2022).

The appearance of the two typical subtypes of DFG, GT and GI, in clinical practice has significant differences and is easy to distinguish and diagnose, as shown in Figure 2A. They are respectively suitable for two different TCM treatment methods, namely, clearing heat and removing dampness and promoting blood circulation and removing stasis, achieving good therapeutic effects, such as faster recovery and significant reduction in amputation cases (<10%). However, there is currently a lack of objective biomarkers for the diagnosis of these subtypes, and relying on conventional laboratory indicators such as FIB and CRP for DFG classification would lack specificity and accuracy. In addition, the difficulty in accurately diagnosing early atypical DFG limits the application of TCM in the treatment of DFG. Therefore, exploring their potential molecular mechanisms will help to understand disease mechanisms from a modern medical perspective, search for new therapeutic targets, and carry out subsequently implement effective interventions.

As an important part of systems biology and an emerging omics technology following transcriptomics, genomics, and proteomics (Muthubharathi et al., 2021), metabolomics has special significance in unravelling the complexities of multifactorial and multisystemic disorders. It can provide more sensitive results on small molecules in a cell, tissue, as well as the molecular contents of a whole organism (Odom and Sutton, 2021). A number of platforms including nuclear magnetic resonance (NMR), gas chromatography-mass spectrometry (GC-MS), and liquid chromatography tandem-mass spectrometry (LC-MS) have been applied for metabolomics analyses. Metabonomics research has been applied to the studies of diabetes under the theory of TCM, such as syndrome differentiation of diabetes of “Xu (deficiency)” and “Shi (excess)” syndromes (Wu et al., 2012), metabolomic profiling combined with TCM diagnosis for pre-diabetic subtypes, “Qi-Yin deficiency with dampness” (subtype A) and “Qi-Yin deficiency with stagnation” (subtype B) of “Qi-Yin deficiency.” However, there is still a lack of metabolomics understanding of diabetes complicated with DFG.

**FIGURE 1:**
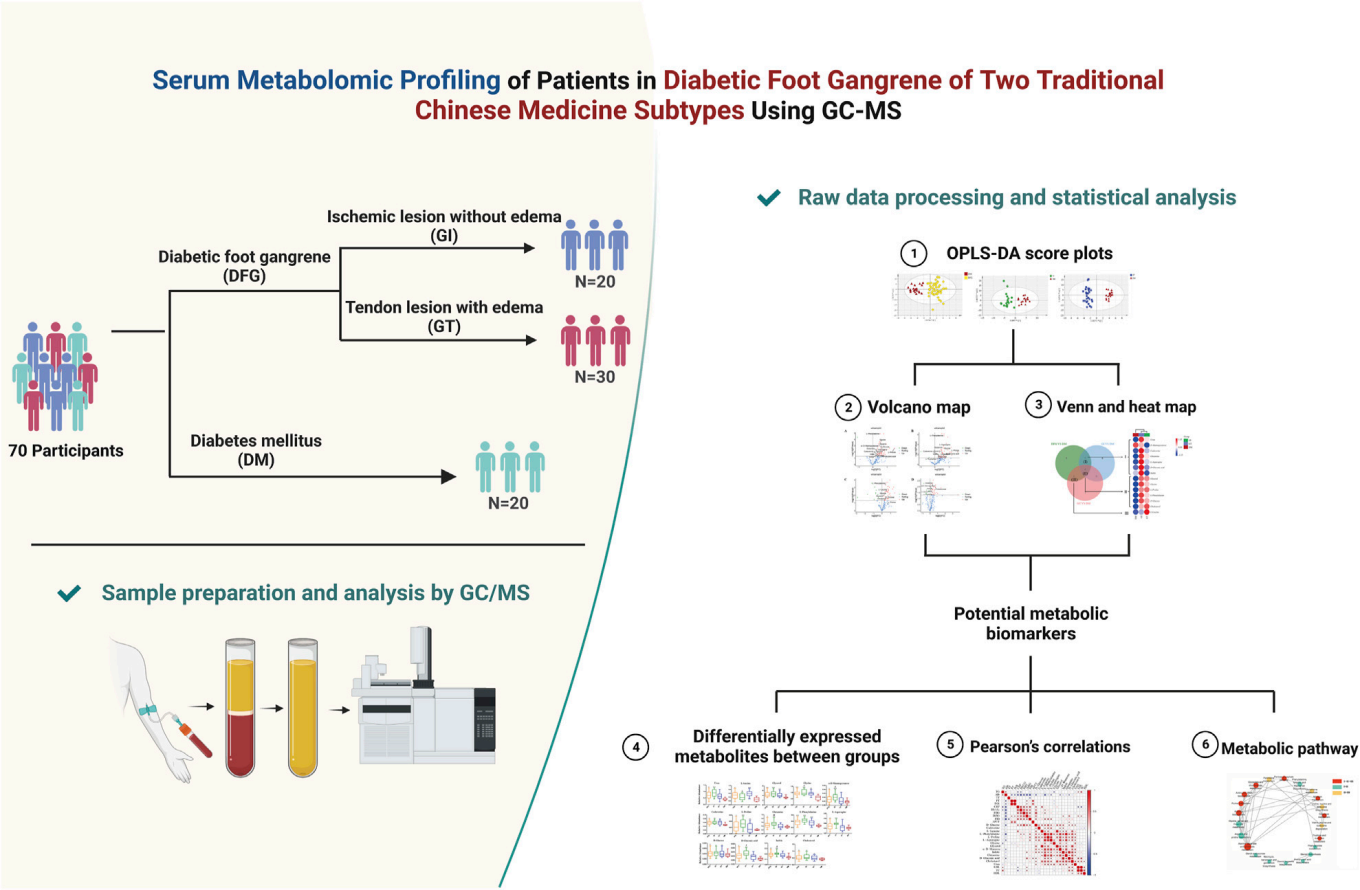
Work scheme of this study.

In this study, a conventional GC-MS followed by orthogonal partial least-squares discriminant analysis (OPLS-DA) were used as untargeted metabolomics approaches to explore the serum metabolomic profiling of 70 participants, including 20 with DM, 50 with DGF (20 with GI and 30 with GT). Kyoto encyclopedia of genes and genomes (KEGG) and MetaboAnalyst were used to identify the related metabolic pathways. Metabolomic profiling can provide insights into the mechanisms underlying the occurrence of DFG, and help us to better understand the impact of different medical strategies on patients. The flowchart of our study is presented in Figure 1.

## 2 Materials and methods

### 2.1 Solvents

Methanol was purchased from Sigma (MO, United States, Lot#WXBC2211V), chloroform and pyridine were purchased from Sinopharm Chemical Reagent limited corporation (Shanghai, China), methoxyamine hydrochloride and N, O-bis (trimethylsilyl) trifluoroacetamide (BSTFA) with 1% trimethylchlorosilane (TMCS) were purchased from Sigma-aldrich (Shanghai, China, Lot#P1890844), heptadecanoic acid was purchased from Aladdin (Shanghai, China, Lot#K1325026). Heptadecanoic acid was used as internal standard.

### 2.2 Patient selection and sample collection

A total of 70 participants, including 30 patients for inclusion in the GT group, 20 for inclusion in the GI group and 20 in the DM group were recruited from Shanghai Traditional Chinese Medicine Integrated Hospital (Shanghai, China) between December 2018 and September 2019. Written informed consents were signed by all participants. The protocol was approved by the Ethics Committee of Shanghai Traditional Chinese Medicine Integrated Hospital (No. 2018-026-1). The diagnosis of DM fulfilled the Standards of Medical Care in Diabetes issued by the American Diabetes Association (2018). The diagnosis of DGF referred to grade 3∼5 in Wagner’s classification of diabetic foot ulcers (Lim et al., 2017). GT patients and GI patients presented diabetic wet gangrene and diabetic dry gangrene independently. More details are available in Supplementary Material.

Fasting venous blood samples were collected from the subjects between 7:00 a.m. and 10:00 a.m. Following collection, whole blood samples were centrifuged for 15 min at 5,000 rpm in a refrigerated centrifuge (4°C), after which the supernatants were aliquoted into eppendorf tubes and stored at −80°C until the subsequent metabolomic analysis.

### 2.3 Sample preparation and analysis by GC/MS

Serum samples were first thawed at room temperature, and prepared following our previously published protocols with minor modifications. A volume of 10 μL of the internal standard solution (heptadecanoic acid methanol solution) was added to 50 μL serum. Added with 200 μL of methanol: chloroform (3:1), and the mixture was shaken for 30 s. The mixture was placed in a refrigerator at −20°C for 10 min, and then centrifuged for 10 min (13,000 rpm, 4°C). Subsequently, 200 μL of supernatant was taken into the injection bottle and dried under nitrogen gas at 40°C. The dried supernatant was combined with 50 μL of methoxyamine hydrochloride in pyridine (15 mg/mL), and the mixture was then vortexed for 30 s and shaken at 30°C for 90 min in a shaker. A volume of 50 μL of BSTFA containing 1% TMCS was added to the sample, and the mixture was shaken for 30 s. Then, the reaction was carried out in an oven at 70°C for 60 min, vortexed for 10 s, and finally allowed to stand at room temperature for 1 h before GC-MS analysis.

A volume of 1 μL derivatized extraction sample was taken for gas-phase mass spectrometry analysis. The injector temperature was set at 260°C. Agilent J&W DB-5ms Ultra Inert 30 m × 0.25 mm, 0.25 µm. The carrier gas was high-purity nitrogen at a constant flow rate of 1.0 mL/min. The initial temperature was maintained at 80°C for 2 min, increased to 290°C and maintained it for 10 min. Subsequently, ion source temperature and quadrupole temperature were 230°C and 150°C, respectively, the solvent delay is 7 min. In the full scan mode (m/z 30–550), the measurements were made by using electron impact ionization (70 eV).

After the raw data was converted into Net-CDF format by the Agilent MSD, it is imported into the R 2.13.2 (Lucent Technologies) and processed. After baseline correction, peak identification, peak alignment and other calculation processes, a three-dimensional matrix table consisting of the specified peak index, sample name, and peak area was finally obtained, and imported into Simca-P 11.5 software (Umetrics, Umeå, Sweden) for multi-dimensional statistical analysis.

### 2.4 Raw data processing and statistical analysis

Principal component analysis (PCA) plot scores were used to describe overall trends or sample clusters. Subsequently, OPLS-DA was used to strengthen the classification among groups and the method of extraction and discrimination of metabolic ions. The metabolic pathways involved in the differential metabolites were analyzed and identified by MetaboAnalyst 5.0 online website, Human Metabolome database, and Kyoto Encyclopedia of Genes and Genomes (KEGG). Statistical product and service solutions (SPSS) 24.0 (IBM, United States) was used for analysis. A *p*-value < 0.05 was considered statistically significant. Demographic and laboratory data were expressed as mean ± SD or median and interquartile range (IQR). Analysis of variance and the t-test were performed on continuous variables of the normal distribution, and the Kruskal-Wallis test was performed on the other continuous variables. Pearson correlation analysis was used to evaluate the correlation between altered metabolites and demographic and laboratory data.

## 3 Results

### 3.1 Clinical and biochemical characteristics of patients

A total of 70 participants were recruited, including 20 with DM, 50 with DGF (20 with GI and 30 with GT). The characteristics of the 70 subjects are summarized in Table 1.

**TABLE 1:** Characteristics of subjects enrolled in the study including DFG, GI, GT and DM.

	DFG	GI	GT	DM
Number of subjects	50	20	30	20
Gender (male/female)	35/15	15/5	20/10	10/10
Age (years); mean (SD)	62.88 (9.51)	66.50 (1.68)	60.47 (1.84)	61.15 (2.41)
<60	17	4 (20.00%)	13 (43.33%)	7 (35.00%)
≥60	33	16 (80.00%)	17 (56.67%)	13 (65.00%)
BMI (kg/m^2^); median (IQR)	23.21 (22.05, 24.22)	23.75 (22.86, 25.03)	22.86 (21.85, 24.22)*	26.04 (22.40, 27.75)
<24	31	12 (42.31%)	19 (54.22%)	7 (35.00%)
≥24	19	8 (57.69%)	11 (43.37%)	13 (65.00%)
WBC (10^9^ L^−1^); median (IQR)	7.00 (5.60, 9.05)	6.70 (5.53, 8.78)	7.10 (6.15, 10.50)	6.20 (5.80, 6.58)
NE (%); mean (SD)	66.89 (10.87)	66.17 (7.23)	67.39 (12.90)	61.92 (10.04)
Hb (g/L); median (IQR)	114.00 (94.00, 121.00)**	115.50 (93.25, 126.80)	112.00 (94.00, 121.00)**	131.00 (118.80, 143.50)
Alb (g/L); mean (SD)	32.36 (5.31)***	33.90 (5.41)*	31.33 (5.07)***	38.15 (3.69)
CRP (mg/L); median (IQR)	100.00 (7.00, 148.00)***^#^	6.50 (3.25, 22.75)^###^	133.00 (110.00, 162.80)***	2.00 (1.00, 2.75)
PT (s); median (IQR)	11.05 (10.50, 12.30)*	10.70 (10.40, 11.35)	11.50 (10.78, 12.70)**	10.60 (10.03, 10.90)
INR; median (IQR)	1.03 (0.97, 1.14)	0.99 (0.96, 1.05)^#^	1.06 (1.00, 1.17)**	0.98 (0.93, 1.01)
APTT (s); median (IQR)	30.50 (29.38, 33.33)	30.60 (28.43, 34.48)	30.50 (29.75, 33.23)	29.15 (27.73, 30.78)
FIB (g/L); median (IQR)	4.35 (3.59, 5.10)	4.35 (3.74, 4.97)	4.34 (3.56, 5.25)	3.02 (2.73, 3.50)
TT (s); median (IQR)	16.15 (15.05, 16.90)	15.80 (14.65, 16.40)	16.35 (15.43, 17.10)	16.00 (15.40, 16.75)
FBG (mmol/L); median (IQR)	7.60 (6.00, 10.63)	6.50 (5.13, 8.00)^#^	9.450 (6.35, 14.18)	7.20 (5.65, 8.78)
2hBG (mmol/L); median (IQR)	9.20 (7.78, 11.60)^###^	9.20 (7.78, 11.60)^##^	14.85 (11.48, 18.43)	11.65 (9.08, 15.00)
HbA1c (%); median (IQR)	7.30 (6.70, 8.48)^##^	7.30 (6.70, 8.48)^##^	10.85 (8.48, 11.70)*	7.60 (7.10, 8.65)
TC (mmol/L); median (IQR)	3.75 (3.27, 4.13)***	3.87 (3.32, 4.38)*	3.65 (3.10, 4.11)***	4.42 (4.09, 5.13)
TG (mmol/L); median (IQR)	1.27 (0.89, 2.77)	1.27 (0.89, 2.77)	1.30 (0.89, 1.56)	1.88 (1.18, 4.07)
HDL (mmol/L); mean (SD)	0.95 (0.27)	1.00 (0.30)	0.92 (0.24)	1.00 (0.22)
LDL (mmol/L); median (IQR)	2.14 (1.72, 2.56)**	2.25 (1.72, 2.78)	2.09 (1.60, 2.44)**	2.90 (2.46, 3.08)

Note: *p < 0.05, compared with the DM group; **p < 0.01, compared with the DM group; ***p < 0.001, compared with the DM group; ^#^ *p* < 0.05, compared with the GT group; ^##^ *p* < 0.01, compared with the GT group; ^###^ *p* < 0.001, compared with the GT group. Reference ranges: WBC: 3.5–9.5 10^9^ L^−1^, NE: 40.0%–75.0%, Hb: male 130–175 g/L, female 115–150 g/L, Alb: 40–55 g/L, CRP: < 10 mg/L, PT: 9.4–12.5 s, INR: 0.88–1.15, APTT: 25.1–36.5 s, FIB: 2.38–4.98 g/L, TT: 10.3–16.6 s, FBG: 3.33–5.55 mmol/L, 2hBG: < 7.78 mmol/L, HbA1c: 4%–6%, TC: < 5.18 mmol/L, TG: 0–1.7 mmol/L, HDL: 0.8–1.8 mmol/L, LDL: 0–3.37 mmol/L.

**FIGURE 2:**
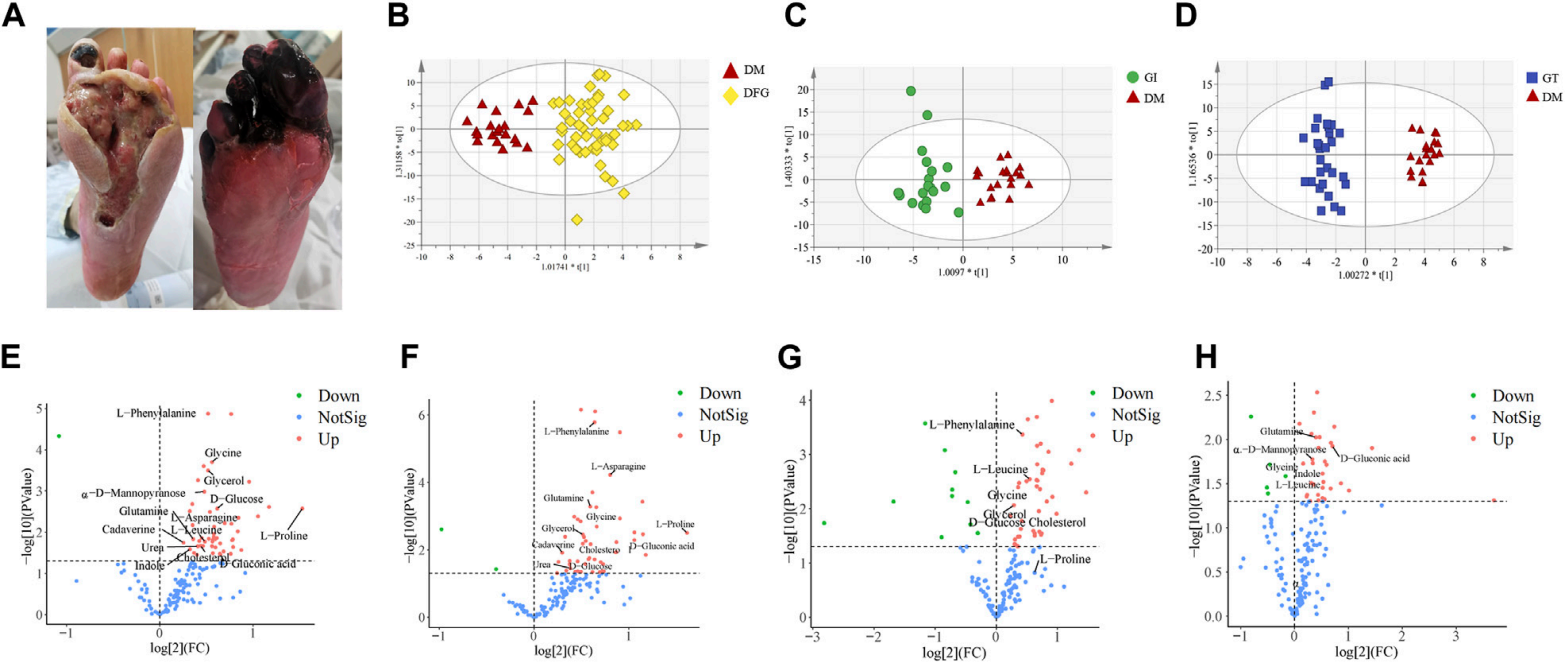
Clinical typical photographs of DFG subtypes, OPLS-DA score plots and volcano plots. **(A)** The two subtypes of DFG, GT (right) and GI (left), belong to Grade IV in Wagner’s Classification (I∼V). **(B)** OPLS-DA score plot of the DFG and DM. **(C)** OPLS-DA score plot of the GI and DM. **(D)** OPLS-DA score plot of the GT and DM. **(E)** Volcano plot of differential metabolites between the DFG and DM groups. **(F)** Volcano plot of differential metabolites between the GI and DM groups. **(G)** Volcano plot of differential metabolites between the GT and DM groups. **(H)** Volcano plot of differential metabolites between the GI and GT groups. The red triangles represent the DM group, the blue squares represent the GT group, and the green circles represent the GI group. For VIP > 1 and *p* < 0.05, the compounds were considered to have statistical significance. Red dots represent upregulated metabolites, green dots represent downregulated metabolites, and blue dots represent NotSig.

Typical clinical photographs of patients in the GI and GT groups was taken from Shanghai Traditional Chinese Medicine Integrated Hospital. In GT group, the great toe and metatarsal of the affected foot showed high tension swelling, accompanied by advanced inflammatory manifestations, manifested as redness, burning, necrosis, purulent blood secretion exuding from the center of the skin lesion, and often had a foul odor. The affected foot might develop a sinus tract or multiple penetrating ulcers on the sole, toes or ankle (Figure 2A). In the GI group, patients showed significant ischemic signs, such as pale and cyanotic toes and metatarsals, progressive ecchymosis on one or multiple toe tips, accompanied by infection and rapid progression of occasional dry necrosis (Figure 2A).

According to Table 1, there were no significant differences in gender, age, white blood cell count (WBC), neutrophil percentage (NE), activated partial thromboplastin time (APTT), fibrinogen (FIB), thrombin time (TT), triglyceride (TG) and high-density lipoprotein (HDL) among DFG, GI, GT and DM groups (*p* > 0.05). Compared with the DM group, the levels of glycosylated hemoglobin (HbA1c) and international normalized ratio (INR) were significantly increased in the GT group (*p* < 0.05), the levels of prothrombin time (PT) was significantly increased in the DFG and GT groups (*p* < 0.05), the levels of C-reactive protein (CRP) was significantly higher in the DFG, GI and GT groups (*p* < 0.05). However, compared with the DM group, the level of body mass index (BMI) was significantly lower in the GT group (*p* < 0.05), the level of hemoglobin (Hb) was lower in the DFG and GT groups (*p* < 0.01), the levels of total cholesterol (TC), low-density lipoprotein (LDL), albumin (Alb) were lower in the DFG, GI and GT groups (*p* < 0.05). In GT group, the levels of INR, fasting blood glucose (FBG), 2-h postprandial blood glucose (2hBG), and HbA1c were significantly increased compared to GI group (*p* < 0.05). However, no significant differences in indexes of BMI, CRP, TC, TG, HDL and LDL were observed between GI and GT groups (*p* > 0.05).

### 3.2 Metabolomic features

Non-target metabolomic profiling was acquired by GC-MS analyzing serum samples collected from all participants. Multivariate data analysis was further performed to obtain more detailed analysis of metabolic differences between groups. OPLS-DA analysis was initially accomplished to obtain the separation trend of DFG from DM. After data preprocessing, OPLS-DA score plots were constructed to determine whether the metabolomic profiles differentiated between groups (Figures 2B–D). In OPLS-DA score plots, R^2^X, R^2^Y and Q^2^ were used to assess the optimizing level, fitness and prediction ability of the model, respectively. In Figure 2B, R^2^X (cum) = 0.519, R^2^Y (cum) = 0.908, Q^2^ (cum) = 0.466. In Figure 2C, R^2^X (cum) = 0.36, R^2^Y (cum) = 0.864, Q^2^ (cum) = 0.43. In Figure 2D, R^2^X (cum) = 0.463, R^2^Y (cum) = 0.963, Q^2^ (cum) = 0.728. There was a good distinction among the groups in all samples, indicating that the endogenous substances in the DFG patients (GI and GT patients) have significantly changed compared with the DM group (Figures 2B, C).

### 3.3 Potential metabolic biomarkers identification and their related metabolic pathways

#### 3.3.1 Potential metabolic biomarkers

OPLS-DA model analysis showed that the serum metabolic profiles of the three groups had significant changes. According to the variable important in the projection (VIP) of the differential metabolites, compounds with VIP > 1 and *p* < 0.05 were screened for differential metabolites.

Volcano plot is generally utilized to present significantly changed metabolites in metabolomic technique. The differential metabolites were identified using a cut-off of Log_2_FC > 1.2 or Log_2_FC < 0.83 and *p*-value < 0.05. The trends of upregulation and downregulation are represented by scatter color, red colored data points represent significantly increased metabolites, green colored data points represent significantly decreased metabolites, and gray blue colored data points represent metabolites with no significant difference. Comparing the metabolic profiles of the DFG and DM groups, and referring to HMDB, KEGG, and other databases, a total of 14 different metabolites were identified, namely, urea, L-leucine, glycerol, glycine, α-D-mannopyranose, cadaverine, L-proline, glutamine, L-phenylalanine, L-asparagine, D-glucose, D-gluconic acid, indole and cholesterol (Figure 2E). These metabolites showed a significant upward trend at the DFG group level. Compared with the DM group, GI group had 13 differential metabolites, including urea, glycerol, glycine, α-D-mannose, cadaverin, L-proline, glutamine, L-phenylalanine, L-asparagine, D-glucose, D-gluconic acid, indole and cholesterol (Figure 2F). Compared with the DM group, the GT group identified a total of seven different metabolites, namely, L-leucine, glycerol, glycine, L-proline, L-phenylalanine, D-glucose and cholesterol (Figure 2G). Six differential metabolites were identified between GI and GT, among which glycine, α-D-mannopyranose, glutamine, D-gluconic acid and indole showed higher levels in GI group, while L-leucine was higher in GT group (Figure 2H).

**FIGURE 3:**
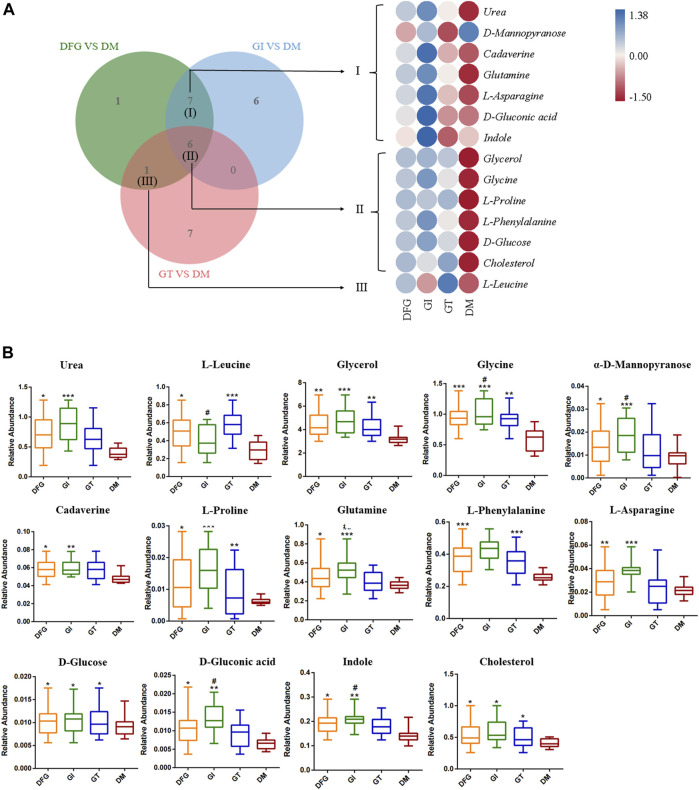
The VENN diagrams, heat maps and box plots of potential metabolic biomarkers. **(A)** The VENN diagrams and heat maps in DFG group, GI group, GT group and DM group. The Venn diagram presents the number of differential metabolites that were changed in the DFG, GT and GT groups compared to the DM group. The heat map presents the differential metabolites between DFG group vs. DM group, GI group vs. DM group, GT group vs. DM group. **(B)** Box plots of potential metabolic biomarkers according to their relative abundance. *p < 0.05, compared with the DM group; **p < 0.01, compared with the DM group; ***p < 0.001, compared with the DM group; ^#^ *p* < 0.05, compared with the GT group; ^##^ *p* < 0.01, compared with the GT group.

We showed the change trend of differential metabolites among groups and classified the upregulated and downregulated metabolites for serum samples by a heat map. In addition, we constructed a Venn diagram combined with the heat map to analyze the metabolite changes in different groups more systematically (Figure 3A). Among these differential metabolites, six shared by two subtypes (GI and GT), and their levels were elevated in both isoforms (Table2; Figure 3AⅡ). These six sharing metabolites were glycerol, glycine, L-proline, L-phenylalanine, D-glucose, and cholesterol. Seven differential metabolites of GI could be regarded as specific potential metabolic markers of GI, including urea, α-D-mannose, cadaverine, glutamine, L-asparagine, D-gluconic acid, and indole (Table2; Figure 3AⅠ). L-leucine can be regarded as a potential metabolic marker of GT (Table2; Figure 3AⅢ). Moreover, we visualized the potential metabolic biomarkers described above based on their relative abundance and plotted the corresponding box plots (Figure 3B).

**TABLE 2:** Potential metabolic biomarkers among the group of DFG (GI, GT) and DM.

No.	RT (min)	Metabolites	DFG vs. DM			GI vs. DM			GT vs. DM			GI vs. GT				
			P	VIP^a^	FC^b^	P	VIP^a^	FC^b^	P	VIP^a^	FC^b^	P	VIP^a^		FC^b^	
1	11.32	Urea	0.022	1.02	1.33	3.96E-06	1.01	2.13	—							
2	11.76	L-Leucine	0.017	1.32	1.40	—			6.70E-08	1.41	1.98	0.026		1.29		0.89
3	11.84	Glycerol	0.002	1.68	1.27	0.001	1.30	1.35	0.009	1.62	1.21					
4	12.55	Glycine	0.001	1.60	1.33	0.001	1.63	1.40	0.003	1.25	1.27	0.042		1.10		1.17
5	13.79	α-D-Mannose	0.019	1.22	1.57	0.001	1.91	1.96	—			0.019		1.19		1.32
6	16.41	Cadaverine	0.018	1.22	1.19	0.010	1.01	1.23	—							
7	19.80	L-Proline	0.027	1.30	1.83	8.15E-07	1.39	2.27	0.0050	1.31	1.45					
8	20.33	Glutamine	0.015	1.44	1.28	0.001	1.49	1.50	—			0.009		1.47		1.32
9	20.39	L-Phenylalanine	0.000	2.27	1.43	8.14E-07	1.93	1.56	4.29E-04	1.55	1.33					
10	21.41	L-Asparagine	0.007	1.47	1.50	4.24E-05	1.77	1.74	—							
11	26.27	D-Glucose	0.033	1.19	1.56	0.029	1.16	1.63	0.026	1.27	1.51					
12	28.16	D-Gluconic acid	0.035	1.26	1.57	0.006	1.19	2.04	—			0.012		1.36		1.63
13	29.84	Indole	0.026	1.22	1.25	0.007	1.29	1.41	—			0.031		1.29		1.24
14	42.11	Cholesterol	0.021	1.21	1.35	0.011	1.27	1.53	0.037	1.13	1.22					

^a^ VIP value was obtained from OPLS-DA.

^b^ Fold change (FC) was calculated as the ratio of the average relative level between two groups.

#### 3.3.2 Associations between the altered metabolites and the biochemical indicators

**FIGURE 4:**
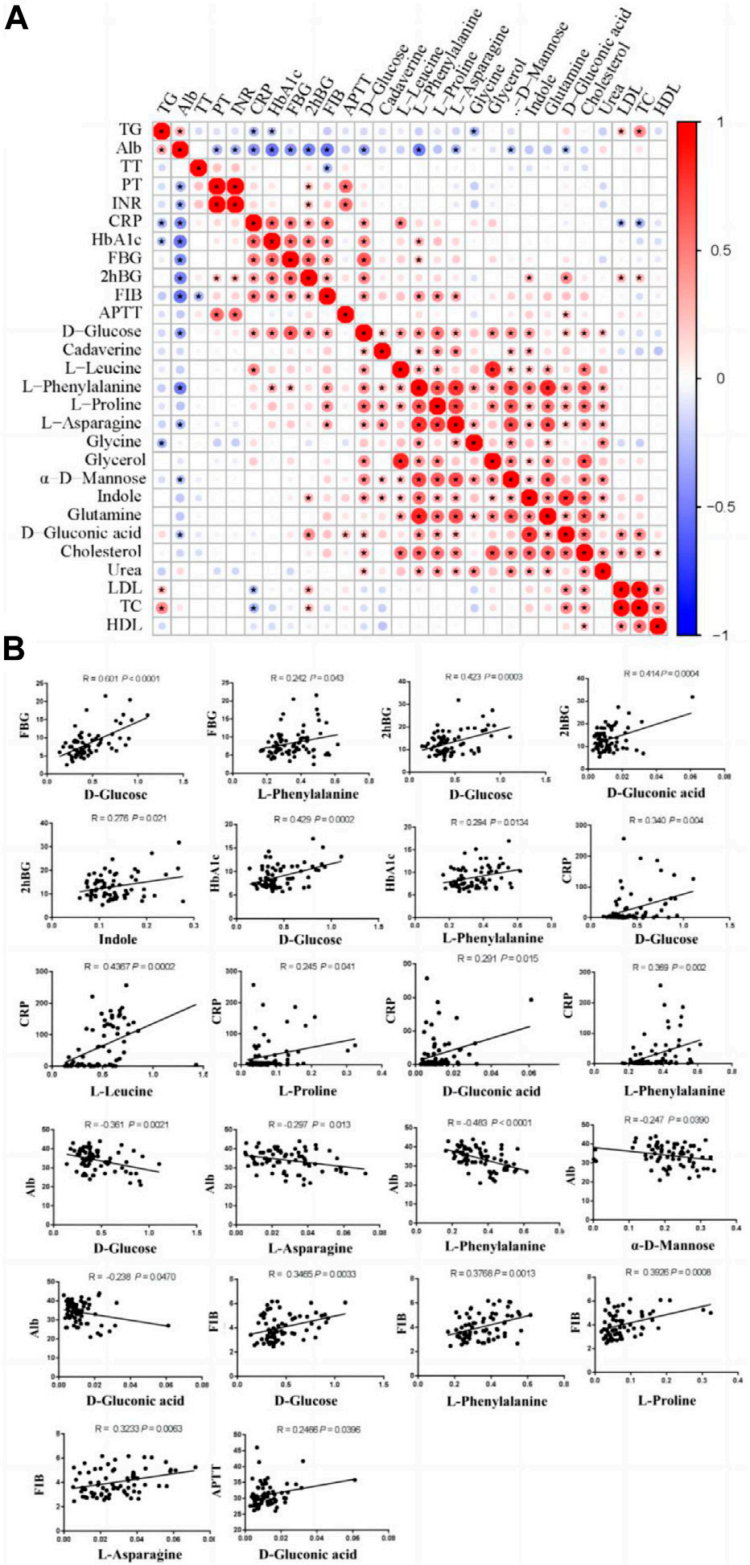
Pearson’s correlations between altered metabolites and biochemical indicators. **(A)** Correlation analyses between biochemical indicators and altered metabolites. **(B)** Positive/negative correlations between biochemical indicators and altered metabolites. FBG, fasting blood glucose; 2hBG, 2 h blood glucose; HbA1c, glycosylated hemoglobin; CRP, C-reactive protein; Alb, albumin; FIB, fibrinogen; APTT, activated partial thromboplastin time.

To further investigate the potential metabolic biomarkers, we conducted Pearson’s correlation analysis between the altered metabolites and biochemical indicators (Figures 4A, B). The results show that D-glucose and L-phenylalanine were positively correlated with the FBG. D-glucose, D-gluconic acid, and indole were positively correlated with 2hBG. D-glucose and L-phenylalanine were positively correlated with HbA1c. In addition, D-glucose, D-gluconic acid, L-proline, L-phenylalanine and L-leucine were positively correlated with CRP. D-glucose, L-asparagine, L-phenylalanine, α-D-mannose, and D-gluconic acid has negative correlations with Alb. D-glucose, L-phenylalanine, L-proline and L-asparagine were positively correlated with FIB. D-gluconic acid was positively correlated with APTT.

#### 3.3.3 The alterations in metabolic pathways of DFG

**FIGURE 5:**
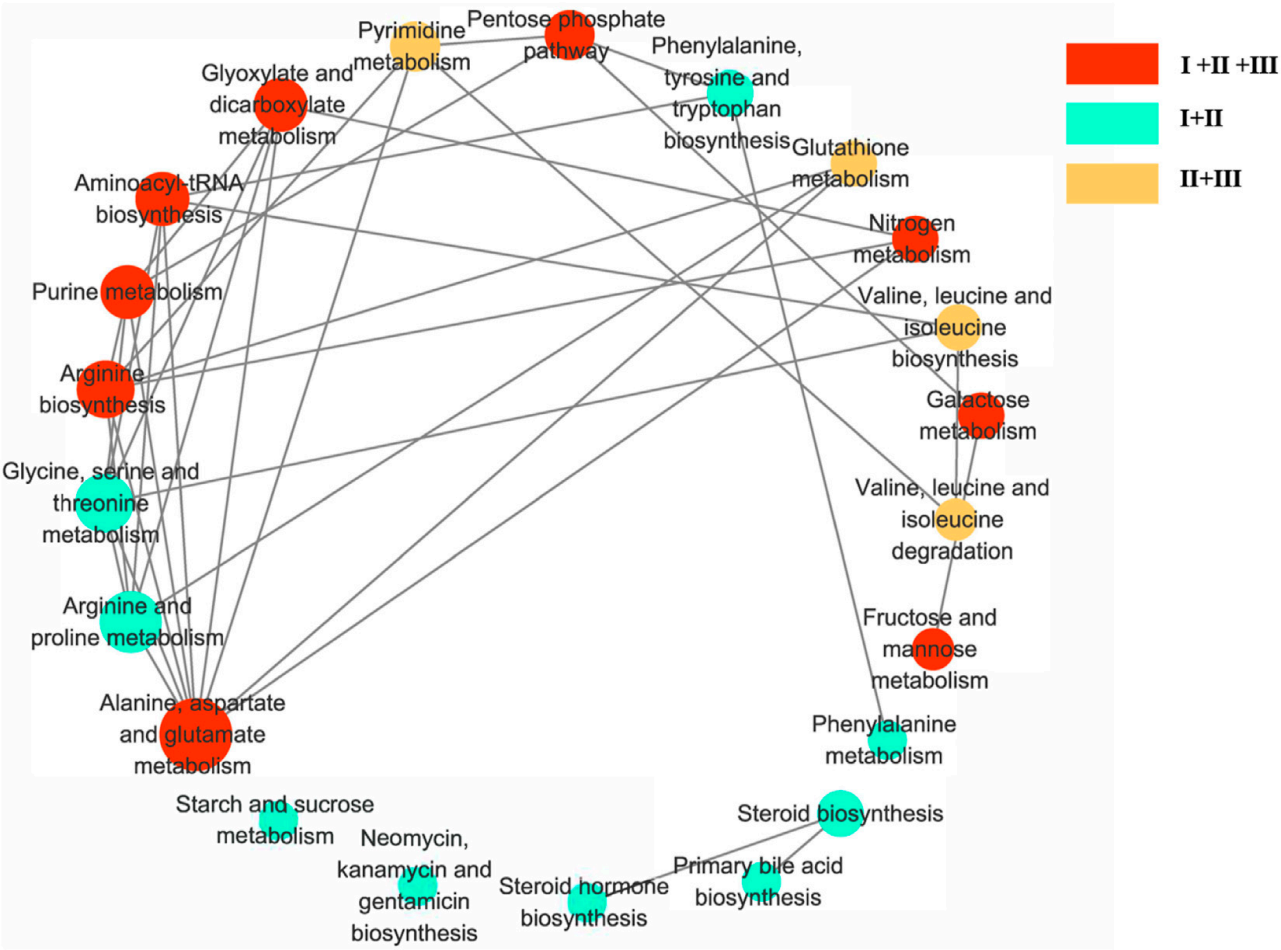
Diagram of metabolic pathway interaction network. Involvement of metabolic pathways with differential metabolites in clusters I–III. The pathway analysis was performed with Kyoto Encyclopedia of Genes and Genomes (KEGG) database (http://www.kegg.jp/kegg) and the picture was generated by Cytoscape. The bigger node represents more pathways connection. Red nodes represent clusters I to III, green nodes represent pclusters I to II, and yellow nodes represent clusters II to III.

MetaboAnalyst 3.0 and KEGG were used to identify the disturbed metabolic pathways. Figure 5 showed the interaction network diagrams of these disturbed metabolic pathways through Cytoscape software. Alanine, aspartate, and glutamate metabolism was the metabolic pathway related to the most of other metabolic pathways, followed by arginine and proline metabolism, arginine biosynthesis, glycine, serine and threonine metabolism, glyoxylate and dicarboxylate metabolism, purine metabolism and aminoacyl-tRNA biosynthesis. Pathways of alanine, aspartate and glutamate metabolism, arginine biosynthesis, purine metabolism and aminoacyl-tRNA biosynthesis, etc., were enriched with metabolites from clusters I to III. pathways of arginine and proline metabolism, glycine, serine and threonine metabolism, phenylalanine, tyrosine and tryptophan biosynthesis, etc., with metabolites from clusters I to II, as well as pathway of pyrimidine metabolism, glutathione metabolism, valine, leucine and isoleucine biosynthesis, valine, leucine and isoleucine degradation with metabolites from clusters II to III.

## 4 Discussion

Diabetic foot (ulceration, infection, gangrene) is one of the most disabling complications of diabetes mellitus (Korzon-Burakowska and Dziemidok, 2011). Generally, diabetic ulcers are prone to develop deep into gangrene, leading to severe infection that cannot be controlled and requires immediate amputation of the affected limb (Carro et al., 2020). According to the clinical syndrome differentiation of TCM, DFG can be divided into dry and wet, which coincides with the understanding of DFG in Western medicine. In the theory of TCM, Qi is the most basic concept, referring to the extremely fine substances with strong vitality in the human body, it is one of the basic substances that make up the human body and maintain the activity of life. Qi deficiency is the most common syndrome, which refers broadly to physical weakness, pallor, shortness of breath, limb weakness, dizziness, and a weak voice (Hou et al., 2022). Based on the syndrome differentiation in the holistic perspective of TCM, the patients with dry gangrene have the clinical manifestations of Qi deficiency and blood stasis syndrome, and local ischemic necrosis without edema, that is called GI. All kinds of examinations have confirmed occlusive changes of limb artery stenosis, often showing abnormalities in coagulation function, such as FIB. While diabetic wet gangrene is mostly characterized by tendon necrosis accompanied by edema, that is called GT. Systemic symptoms such as high fever, nausea and vomiting may occur, and local inflammatory reactions may occur, accompanied by elevated biochemical indicators such as CRP. And the corresponding TCM treatments achieved better therapeutic effects, that the clinical amputation rate of less than 10%. In order to enhance our understanding of the disease, this study applied the GC/MS based metabolomics method to explore the serum metabolomic profiling among patients with DFG in different TCM subtypes, which is conducive to the cognition of the pathogenesis of DFG in different subtypes, and thus helps to improve the strategies in diagnosis and treatment.

A large number of clinical trials and basic research have shown that diabetes is not only a metabolic disease characterized by hyperglycemia, but also an inflammatory disease (King, 2008). The levels of many inflammatory factors were significantly increased, especially CRP, TNF-α and IL-6. These inflammatory factors can activate a series of signaling pathways and cause the occurrence of chronic complications of diabetes (Dandona et al., 2004). Clinical studies have found that patients with GT still have microinflammation even though the wound is healed, which is one of the important reasons for the recurrence of GT. Inflammatory reaction runs through the whole process of occurrence and development of GT. When the body is infected or injured, CRP plays an important role in the immune process of the body and increases sharply, which may activate complement and strengthen phagocytosis of macrophages (Zhang et al., 2021). It has been concluded that CRP can be used as an effective indicator to identify the degree of infection between DFG patients and patients with different severity of foot ulcers, and can directly reflect the level of inflammation (Wu et al., 2023). Our study found that CRP levels were significantly higher in GT patients, suggesting that patients with GT have manifestations of infection, higher levels of inflammation, and poorer vascular endothelial function.

Albumin is abundant in human blood, and research has identified a wide range of putative roles for the protein in modifying inflammation, maintaining vascular endothelial integrity and acid-base balance, and ligating endogenous and exogenous compounds (Ferrer et al., 2018). Albumin can offer protection from inflammatory processes and the associated damage to microcirculation and tissues (Ferrer et al., 2018). Preexisting hypoalbuminemia is a prognostic indicator of worse outcome for a broad spectrum of diseases in both medical (Fulks et al., 2010; Pavliša et al., 2017). There is a close correlation between the increase of CRP and the decrease of serum albumin levels. For instance, in patients undergoing elective open colorectal surgery, it has been reported that preoperative CRP was useful in predicting the development of hypoalbuminemia on postoperative days 3 and 7 (Sonoda et al., 2015). Hyperglycemia is the most important factor affecting the occurrence and development of DFG (Burgess et al., 2021). Long-term hyperglycemia will lead to metabolic disorders, dehydration of tissues, organs and cells, and reduced immune function, increasing the risk of lower limb amputations (Boulton et al., 2008). Good glycemic control has been reported to delay the progression of DFG and reduce the risk of amputation by 36% (Burgess et al., 2021). HbAlc is regarded as a risk factor for the development of DFG, because it can promote inflammatory response, peripheral neuropathy, microvascular complications, and decrease in related cell growth factors. FBG and 2hBG can indirectly reflect the body’s own basal insulin secretion and evaluate the level of islet B cells (Savage et al., 2007). The levels of FBG, 2hBG, and HbA1c in the GT group were higher than those in the other two groups, suggesting an increased risk of lower limb amputation due to long-term poor glycemic control in diabetic patients with GT.

In addition, T2DM is often accompanied by dyslipidemia. In this study, the levels of TC, TG, HDL and LDL were no significantly different from the reference levels in clinical diagnosis (TC: < 5.18 mmol/L, TG: 0–1.7 mmol/L, HDL: 0.8–1.8 mmol/L, LDL: 0–3.37 mmol/L, respectively). TG was significantly elevated in patients with DM, but the levels of TC, HDL and LDL were no significantly different from the reference levels in clinical diagnosis (<5.18 mmol/L, 0.8–1.8 mmol/L and 0–3.37 mmol/L, respectively). Diabetes is a disease of impaired glucose metabolism as well as disturbed lipid metabolism. Diabetes affects almost all lipids and lipoproteins, and chronic dyslipidemia is common in diabetic patients. For diabetic foot, controversial results have been previously reported. An association (Al-Shammaree et al., 2017) was reported between serum TG and the progression of diabetic foot ulcer in T2DM patients, whereas in some other studies, no association was found between hypercholesterolemia and diabetic foot ulcer progression (Al-Shammaree et al., 2017). Conversely, hypercholesterolemia was also reported as a risk factor for the development of ulcers. Our research suggests that hypercholesterolemia may not be a determinant of DFU progression in T2DM patients (Atosona and Larbie, 2019).

Serum non-targeted metabolomic profiling was analyzed using a chemometric approach, identifying the differential metabolites between DM and DFG. Among these differential metabolites, six were shared by two subtypes of GI and GT with elevated levels, which were glycerol, glycine, L-proline, L-phenylalanine, D-glucose, and cholesterol. The specific differential metabolites of each subtype were assumed as their potential metabolic biomarkers, which were seven for GI, and one for GT (Figure 5). The potential metabolic biomarkers of GI were urea, α-D-mannose, cadaverine, glutamine, L-asparagine, D-gluconic acid, and indole, as well as L-leucine for GT. The differential metabolites of DFG are mainly involved in pathway of alanine, aspartate, and glutamate metabolism, which is the most important metabolic pathway as it related to the most of other metabolic pathways, followed by arginine and proline metabolism, arginine biosynthesis, glycine, serine and threonine metabolism, glyoxylate and dicarboxylate metabolism, purine metabolism and aminoacyl-tRNA biosynthesis.

As one of the potential metabolic biomarkers for GI, glutamine is the precursor of nitrogen-containing substances such as peptides, proteins and neurotransmitters in the body, as well as an important carbon source for gluconeogenesis and glycogen synthesis (Tapiero et al., 2002). It may reduce the production of pro-inflammatory cytokines in human intestinal mucosa via a post-transcriptional pathway (Tapiero et al., 2002) and contribute to modulate the inflammatory state due to cytokine imbalance. Meanwhile, metabolomics studies have reported glutamine is associated with increasing oxygen free radicals (Yang et al., 2021) and promoting vasoconstriction as a potential biomarker for blood stasis evidence, which may be consistent with the “blood stasis” syndrome of GI. Indole is one of the metabolic products of tryptophan transformation by gut microbiota (Su et al., 2022). Indole stimulates GLP-1 secretion from intestinal L cells, resulting in insulin release and reduced blood glucose levels. The levels of α-D-mannose and D-gluconic acid in GI were higher than GT, which are related to glucose metabolism. Gluconic acid is related to hyperglycemia and oxidative stress, and is closely related to the occurrence of DFG. Uric acid affects the occurrence and development of diabetes and its complications through pathological mechanisms such as inflammation, oxidative stress, vascular endothelial injury, and inhibition of insulin signaling pathways (Xiong et al., 2019). A regression analysis suggested that high serum uric acid level was a significant and independent risk factor for diabetic foot ulcers in Chinese women with type 2 diabetes. In addition, some studies have suggested that foot deformity and serum urea are associated with diabetic lower extremity amputations (Xiong et al., 2019). Available data indicate that certain inactivated tissues can produce biologically active polyamines, in particular putreghine and cadaverine, in the presence of bacteria (Shah and Swiatlo, 2008). In addition to the loss of protein in the wound itself (Daigle et al., 2013), the phenomenon of emaciation, anemia, and hypoproteinemia in a short period of time in patients with common chronic ulcers may also be related to the absorption of a large number of polyamines in local necrotic tissue into the blood, inducing systemic inflammatory response and causing high body consumption (Iizaka et al., 2010). Asparagine and other biologically active molecules have a vital role in cell catabolism and signaling, host anti-oxidative ability, and immunity under physiological and pathological conditions (Wu, 2013). However, whether asparagine is protective or diabetogenic is still a subject of debate. GI is characterized by blood stasis and presents as ischemic lesions without edema. In the GI subtype, D-gluconic acid and L-asparagine are positively correlated with activated partial thromboplastin time (APTT) and fibrinogen (FIB) as coagulation function indicators. D-gluconic acid and L-asparagine may be more valuable in the diagnosis of GI of blood stasis. These potential metabolic biomarkers are mainly involved in pathways of alanine, aspartate and glutamate metabolism, aminoacyl-tRNA biosynthesis, nitrogen metabolism, arginine biosynthesis, fructose and mannose metabolism, etc.

L-leucine is the potential metabolic biomarker of GT, is one of BCAA mainly involved in the pathway of valine, leucine and isoleucine biosynthesis and valine, leucine and isoleucine degradation, etc. BCAA have long been implicated in the etiology of type 2 diabetes. Blood levels of BCAAs are positively correlated with insulin resistance, obesity, and diabetes in both humans and rodents (Wu, 2013). Further, specifically restricting dietary BCAAs restores metabolic health to Western diet-induced obese (DIO) mice, rapidly normalizing weight, adiposity, and glycemic control without caloric restriction (Batch et al., 2013). Mechanistic target of rapamycin (mTOR) is a central signaling molecule that regulates cell growth and metabolism, as well as a critical regulatory factor of macrophage polarization, which can control the number and function of different types of macrophages according to the basic nutritional state of the body (Connelly et al., 2017). Leucine is considered to be a potent natural activator of mTORC1 and an anabolic trigger of the mTORC1 pathway. The increase of leucine level leads to the over-activation of mTORC1 pathway, increases the mRNA expression of tumor necrosis factor alpha (TNF-α), inducible nitric oxide synthase (iNOS) and interleukin-6 (IL-6), and increases the protein expression of glycolytic enzymes pyruvate kinase M2 (PKM2), HK-2, elevated lactate dehydrogenase A (LDHA), as well as hypoxia and hypoxia-inducible factor-1alpha (HIF-1alpha), Raptor, and CD86, which can release inflammatory factors and promote the polarization of macrophage M1, leading to the prolonged and non-healing of diabetic foot ulcers (Connelly et al., 2017). Clinical studies showed that wound healing in GT patients was still characterized by micro-inflammation, mainly infection, and high inflammation index. Compared with GI group, leucine level was increased in GT group, which is consistent with the elevated inflammatory response in GT group. Clinical studies have found that patients with GT still have microinflammation even though the wound is healed, which is one of the important reasons for the recurrence of GT. Inflammatory reaction runs through the whole process of occurrence and development of GT. CRP is an important indicator of inflammation (Wu et al., 2023). In correlation analysis, we found that leucine was positively correlated with CRP, which further suggested that leucine may be a potential metabolic biomarkers of GT.

In addition, branched-chain amino acids (BCAAs) account for 35% of the total content of essential amino acids in muscle proteins (Rennie and Tipton, 2000). BCAAs, especially leucine, has been reported to promote muscle-protein synthesis. Elderly subjects and those with diabetes or peripheral arterial disease (PAD) (Cummings et al., 2018) are more likely to have sarcopenia, and the long-term presence of a DFU may impair limb activity and might have resulted in a faster loss of lower extremity muscle mass in our population. Nevertheless, there is currently no direct evidence suggesting a correlation between BCAA supplements and wound healing.

Compared with DM, the levels of glycerol, glycine, L-proline, L-phenylalanine, D-glucose, and cholesterol in GI and GT groups were significantly increased. Glycerol and cholesterol indicate the disorder of glucose and lipid metabolism, which might cause the damage of vascular endothelial cells, the change of coagulation function, and accelerated the formation of diabetic foot. Diabetes is characterized by insulin resistance and an imbalance of catabolic/anabolic hormone that affect systemic metabolism (Tripathi and Srivastava, 2006). Insulin resistance can lead to protein degradation, releasing amino acids (Zhang et al., 2022), which are substrates for gluconeogenesis. In our study, the differential metabolites between DFG patients and DM patients were glycine, L-proline and L-phenylalanine. Glycine is a precursor of uric acid, which can be synthesized from serine, inhibits the expression of NF-κB and pro-inflammatory factors in adipose tissue, and is negatively correlated with the development of diabetes mellitus and glucose tolerance abnormalities (Rennie and Tipton, 2000). Meanwhile, glycine is a potential biomarker for blood stasis in coronary heart disease. It has been found that glycine levels are elevated in blood stasis evidence in a variety of diseases (Yi et al., 2021). A related study found that plasma proline increased in the group with coronary heart disease blood stasis evidence and decreased in the group without blood stasis evidence (Jian et al., 2010). Phenylalanine, tyrosine and tryptophan are aromatic amino acids that are biomarkers of diabetes risk, mediated through insulin resistance, which may cause impaired insulin secretion, and a persistent hyperglycemic state that can lead to non-enzymatic glycosylation of some structures, proteins, and nucleic acids, which triggers diabetic foot. Phenylalanine is oxidized to tyrosine by phenylalanine hydroxylase in the body, which participates in the body’s glucose and fat metabolism. Blood phenylalanine concentration leads to an increase in reactive oxygen species in the organism, which may trigger vascular endothelial function damage and an increase in reactive oxygen species, leading to vasoconstriction (Tripathi and Srivastava, 2006) and triggering blood stasis.

The differential metabolites of DFG are mainly involved in pathway of alanine, aspartate, and glutamate metabolism, which is the most important metabolic pathway as it related to the most of other metabolic pathways.

Alanine metabolism is linked to glycaemic control. Expression of alanine aminotransferases is increased in the liver in mice with obesity and diabetes, as well as in humans with type 2 diabetes. Hepatocyte-selective silencing of both alanine aminotransferase enzymes in mice with obesity and diabetes retards hyperglycaemia and reverses skeletal muscle atrophy through restoration of skeletal muscle protein synthesis. Mechanistically, liver alanine catabolism driven by chronic glucocorticoid and glucagon signalling promotes hyperglycaemia and skeletal muscle wasting (Okun et al., 2021). Therefore, abnormal alanine metabolism may be related to the enhancement of muscle catabolism and amino acid imbalance, which may lead to malnutrition and decreased immunity in the context of chronic hyperglycemia, thus aggravating the susceptibility and deterioration of diabetic foot ulcers. Glutamate is the principal excitatory neurotransmitter and energy source of the central nervous system, which can generate α-ketoglutaric acid during oxidative decarboxylation, and further participate in the tricarboxylic acid cycle (TCA cycle) to produce energy (Petroff, 2002). In DFG patients, due to microvascular disease and neuropathy, inadequate blood supply and impaired nerve function in the foot may affect the energy metabolism and nerve conduction of local tissues, exacerbating the occurrence and healing difficulties of foot ulcers.

Abnormal metabolism of these amino acids may aggravate oxidative stress, increase free radical generation, damage vascular endothelial cells, and aggravate vascular lesions in diabetic patients (Cassano et al., 2016; Martínez-Vacas et al., 2021). In addition, their metabolites can also participate in the process of inflammatory response (Wang et al., 2021), which is closely related to the formation and progression of foot ulcers.

Identifying potential metabolic biomarkers means being able to more precisely identify which subtype a patient belongs to, which will greatly improve the targeting of treatment. For example, we found that the differential metabolites of DFG mainly involve the metabolic pathways of alanine, aspartate and glutamate, which suggests that targeted drugs should be used for this pathway. Moreover, potential metabolic biomarkers can be used for diagnosis, as well as to assess the prognosis of the disease and monitor the effect of treatment. For a subtype, high or low levels of a particular metabolite may be associated with a poorer prognosis, or the extent to which it changes with treatment may serve as an indicator of treatment response. Understanding the biomarkers between different subtypes could help scientists design new drugs or therapies that specifically target the specific metabolic pathways that lead to disease onset and progression. Identifying specific metabolic markers of different subtypes of any disease will have a profound impact on personalized medicine, enabling more accurate and effective treatment.

## 5 Conclusion and perspectives

Metabolic spectrum can sensitively reflect various physiological and pathological changes. In addition, it can also clarify the concept of “subtypes” in the complex physiological system of TCM. In conclusion, these potential biomarkers may help to reveal the pathological changes of different subtypes of DFG, objectively and comprehensively understand the disease mechanisms of different subtypes of DFG. In this study, a GC-MS-based metabolomics approach was employed to compare the metabolic characteristics between patients with GI and GT. A total of 14 differential metabolites were identified as potential metabolic biomarkers of DFG, including urea, L-leucine, glycerol, glycine, α-D-mannopyranose, cadaverine, L-proline, glutamine, L-phenylalanine, L-asparagine, D-glucose, D-gluconic acid, indole and cholesterol. Among them, urea, α-D-mannopyranose, cadaverine, glutamine, L-asparagine, D-gluconic acid and indole are related to GI, and L-leucine is related to GT. Our findings demonstrate that a metabolomic approach is a promising tool for exploring the complex metabolic states of diseases, which can help to reveal the pathological changes of different subtypes of DFG and to understand the disease mechanisms of different subtypes of DFG objectively and comprehensively. However, our research has the following limitations. The design of this study was considered exploratory due to the relatively small sample size and the use of a non-targeted methodology. To confirm the results obtained in this study, targeted metabolomics studies in large prospective studies are needed, and the results need to be validated and generalized.

## Figures and Tables

**Table:** 

**2hBG**	2h blood glucose
**AGEs**	advanced glycation end
**Alb**	albumin
**APTT**	activated partial thromboplastin time
**BCAAs**	branched-chain amino acids
**BMI**	body mass index
**CRP**	C-reactive protein
**DFG**	diabetic foot gangrene
**DIO**	diet-induced obese
**FBG**	fasting blood glucose
**FIB**	fibrinogen
**G3PDH**	glucose 3-phosphate dehydrogenase
**GC-MS**	conventional gas chromatography-mass spectrometry
**GI**	ischemic lesion without edema
**GT**	tendon lesion with edema
**HbA1c**	glycosylated hemoglobin
**HIF-1alpha**	hypoxia-inducible factor-1alpha
**IL-6**	interleukin-6
**iNOS**	inducible nitric oxide synthase
**INR**	international normalized ratio
**KEGG**	kyoto encyclopedia of genes and genomes
**LC-MS**	liquid chromatography tandem-mass spectrometry
**LDHA**	lactate dehydrogenase A
**LDL**	low-density lipoprotein
**mTOR**	mechanistic target of rapamycin
**NE**	neutrophil percentage
**NMR**	nuclear magnetic resonance
**OPLS-DA**	orthogonal partial least-squares discriminant analysis
**PAD**	peripheral arterial disease
**PCA**	principal component analysis
**PKM2**	pyruvate kinase M2ROS, reactive oxygen species
**PT**	prothrombin time
**SD**	standard deviation
**SPSS**	statistical product and service solutions
**TC**	total cholesterol
**TCM**	traditional Chinese medicine
**TG**	triglyceride
**TNF-α**	tumor necrosis factor alpha
**TT**	thrombin time
**VIP**	variable important in the projection
**WBC**	white blood cell count

## Data Availability

The data presented in the study are deposited in the jianguoyun repository, the link of the repository is https://www.jianguoyun.com/p/DS2VSLwQiv_EDBjHq74FIAA.
