# Supplementary material for: Applying GC-MS based serum metabolomic profiling to characterize two traditional Chinese medicine subtypes of diabetic foot gangrene
Source: Front Mol Biosci. 2024 Apr 25;11:1384307. doi: 10.3389/fmolb.2024.1384307 (PMC11079259; doi:10.3389/fmolb.2024.1384307)
Supplement: Supplementary file 1 [file DataSheet1.pdf]

Supporting Information

Applying GC-MS Based Serum Metabolomic Profiling to Characterize Two Traditional Chinese Medicine Subtypes of Diabetic Foot Gangrene

Jiawei Feng[1], Yuqing Wang[1], Shengmin Xiang[1], Yun Luo[1], Yongcheng Xu[1], Yuzhen Wang[1], Yemin Cao[1], Mingmei Zhou[2*], Cheng Zhao[1*]

1. Shanghai Traditional Chinese Medicine Integrated Hospital, Shanghai University of Traditional Chinese Medicine, Shanghai 200082, China.

2. Institute of Interdisciplinary Integrative Medicine Research, Shanghai University of Traditional Chinese Medicine, Shanghai, 201203, China.

***** Correspondence:

chengzhao_79@163.com; Tel.: +86-21-65460954 (C.Z.)

zhoumm368@163.com; Tel.: +86-13166300770 (M.Z.)

Diagnostic Criteria

1. The diagnostic criteria of DM

(1) Typical diabetes symptoms and random blood glucose ≥ 11.1 mmol/L; (2) oral glucose tolerance test (OGTT) 2 h blood glucose ≥ 11.1 mmol/L; (3) fasting blood glucose (FBG) ≥ 7.0 mmol/L. Diagnosis can be made if one of the above is met.

2. The diagnostic criteria of DFG

2.1 The diagnosis of DGF referred to grade 3~5 in Wagner's classification of diabetic foot ulcers.

2.2 The diagnosis criteria of DFG subtypes

The DFG subtypes were divided into GT and GI according to the clinical symptoms and are identified by two TCM experts with senior titles. The diagnosis results were included in the study after unification, and if they were inconsistent, the third expert would conduct syndrome differentiation. If they were inconsistent, the cases would not be included.

2.2.1 The diagnostic criteria of GI

2.2.1.1 Clinical symptom

Critical limb ischemia/chronic limb-threatening ischemia (CLI/CLTI), limb pain, intermittent claudication, chronic rest pain. The pain may be worsened in an elevated leg and improved in the dependent position due to compromised blood flow. The area was rubor, pale and raised in the early stage. Capillary refill is reduced, and there may be a loss of overlying hair. Ankle pulses are frequently absent.

2.2.1.2 Physical and chemical examination

All kinds of examinations proved that there was an occlusive change of limb artery stenosis, and color Doppler, CT, DSA, vascular ultrasound, and vascular electro-optical volume flow chart confirmed that there was limb artery stenosis or occlusion; angiography mainly focuses on arterial lesions of lower limbs, and popliteal artery lesions are most common in distal arteries, accounting for more than 80%. The morphology of vascular lesions is similar to arteriosclerosis obliterans. Due to extensive limb arteriosclerosis and diabetes, there are fewer

collateral vessels, and the vessels can be tortuous, narrow, and occluded. The lower limb artery ankle-brachial ratio decreased significantly; plain X-ray film showed obvious calcification shadows in the aortic arch, abdominal aorta, or lower limb artery.2.2.1.3 Blood-stasis syndrome.

Blood-stasis syndrome can be diagnosed when the symptoms conform to 1 main criterion, or 2 secondary criteria.

Main Criteria

(1) Dull red or purple or cyanosed tongue texture or with ecchymoses and petechiae, or cyanosed, purple black, varicose or coarse swelling sublingual vein. (2) Dull red or purple or cyanosed face, lips, gum, periorbital region, and finger/toe-end. (3) Varicosity or telangiectasia at any sites. (4) Blood outside the meridians (causing blood stasis and accumulation in organs, tissues, subcutaneous or serosal cavity). (5) Abdominal tenderness and tightness. (6) Dark menstrual flow, or slightly dark with blood clots. (7) Vascular occlusion or moderate to severe stenosis (50%) showed in imaging examinations. (8) Material evidence of thrombosis, infarction, or embolism.

Secondary Criteria

(1) Fixed pain, or stabbing pain, or pain aggravated at night. (2) Limb numbness or hemiplegia, or joint swelling and deformity. (3) Dry and scaly skin (rough and thick skin with increased scale). (4) Unsmooth, intermittent or hardly perceivable pulse. (5) Pathological lumps, including organomegaly, neoplasm, inflammatory or non-inflammatory masses and tissue hyperplasia. (6) Mild vascular stenosis (<50%) showed in imaging and other examinations. (7) Abnormal results of physicochemical examinations such as hemodynamics, hemorheology, platelet function, coagulation function, fibrinolysis function, microcirculation, chest X-ray and ultrasonography that indicate circulation disorder, abnormal microvascular structure and function, or concentrated, viscous, coagulated and aggregated state of blood. (8) Trauma, operation or abortion within

2.2.2 The diagnostic criteria of GT

2.2.2.1 Clinical symptom

Classical clinical signs of infection such as redness, warmth, swelling, pain in the foot. The affected limb has no obvious ischemic symptoms: ankle-brachial index (ABI) > 0.9, no limb numbness or intermittent claudication, dorsalis pedis (DP) and posterior tibial (PT) artery pulsations were palpable. Deep infection, usual systemic signs of infection (*e.g.*, fever, elevated white blood cell count). Drainage and edema in the setting of a patient with a previous foot ulcer or tissue injury secondary to diabetes.

2.2.2.2 Physical and chemical examination

(1) Diagnostic physical and chemical examination: three high-high blood sugar, high white blood cell, high erythrocyte sedimentation rate, three low-albumin, low red blood cell, low hemoglobin Doppler vascular examination, vascular ultrasound: the blood flow of the dorsal foot artery and the posterior tibial artery of the affected foot is in the normal range, or there is partial stenosis or occlusion.

(2) Auxiliary physical and chemical examination: X-ray examination showed foot abnormalities. The pathological examination of the tendon can be performed under experimental conditions. The degeneration, edema, or necrosis of the tendon can be seen.2.2.2.3 Dampness-heat-related symptoms

Dampness-heat-related symptoms can be diagnosed when the symptoms conform to 1 main criterion, or 2 secondary criteria.

Main Criteria

(1) Slimy yellow tongue fur, thick tongue fur, red tongue; (2) slippery pulse, or rapid-slippery pulse.

Secondary Criteria

(1) Heavy body, obesity, heavy sensation of head; (2) sticky stool with ungratifying defecation, constipation; (3) sticky and greasy in mouth, bitter taste in the mouth, halitosis, dry mouth and thirst; (4) deep-colored urine.

3. Inclusion Criteria

(1) Conform to the diagnostic criteria of Xi's diabetes mellitus gangrene type or ischemic type; (2) there are no patients with severe liver and kidney function injury, mental illness, and other

serious diseases; (3) first foot break; (4) Obtain the consent of the patient or his family; (5) Does not violate the requirements of medical ethics.

4. **Exclusive Criteria**

(1) Skin ulcer caused by electrical, chemical, or radiation injury, tumors, varicose veins, or other reasons; or malignant lesions within the ulcer; (2) severe clinical infection indicated by cellulitis, fever, elevated white blood cell count, bacterial culture, or increased (high-sensitivity) C-reactive protein levels; (3) severe uncontrollable hypertension with systolic blood pressure ≥ 160 mmHg or diastolic blood pressure ≥ 110 mmHg; (4) serum albumin levels < 28 g/L; (5) hemoglobin < 90 g/L; (6) platelet count $< 50 \times 10^9$/L; (7) severe heart, liver, or kidney injury, in case of medical treatment that may seriously affect the safety and treatment; (8) pregnancy, family planning, or breastfeeding women; (9) cognitive dysfunction preventing fully informed consent; (10) allergic disposition or allergic to the ingredients of the treatment under investigation and reference drugs; (11) participation in other clinical trials during the past one month; (12) in the judgment of the researcher, inability to complete the trial or comply with its requirements.
